# Progressive Increase of Matrix Metalloprotease-9 and Interleukin-8 Serum Levels during Carcinogenic Process in Human Colorectal Tract

**DOI:** 10.1371/journal.pone.0041839

**Published:** 2012-07-25

**Authors:** Fiorella Biasi, Tina Guina, Marco Maina, Mario Nano, Alessandro Falcone, Emiliano Aroasio, Giorgio Maria Saracco, Mauro Papotti, Gabriella Leonarduzzi, Giuseppe Poli

**Affiliations:** 1 Department of Clinical and Biological Sciences, University of Turin, San Luigi Gonzaga Hospital, Orbassano (Turin), Italy; 2 Clinical Biochemistry Laboratory, San Luigi Gonzaga Hospital, Orbassano (Turin), Italy; Instituto de Investigación Sanitaria INCLIVA, Spain

## Abstract

**Background:**

Inflammatory reactions, known to promote tumor growth and invasion, have been found associated with colorectal carcinoma (CRC). Macrophages are the chief component of the inflammatory infiltration that occurs early in the progression from non-invasive to malignant tumor, with a switch from the pro-inflammatory phenotype to the tumor-promoting phenotype. Tumor and stroma are additional sources of inflammation-related molecules. The study aimed to evaluate, during colorectal carcinogenesis from benign to malignant phases: i) the trend of serum levels of IL-8, IL-6, TGFβ1, VEGF and MMPs; ii) the parallel trend of CRP serum levels; iii) derangement of the principal TGFβ1 receptors (TGFβ1RI/RII) in tumor tissues.

**Methodology/Principal findings:**

96 patients with colon adenomas or CRC at different stages of progression, and 17 controls, were recruited. Serum IL-8, IL-6, TGFβ1, VEGF, MMPs and CRP levels were analyzed before endoscopy or surgery. TGFβ1 receptors were evaluated in adenoma biopsies and surgically-removed colorectal adenocarcinomas. Serum levels of IL-8 in adenocarcinoma patients were increased from stage II, when also the enzymatic activity of MMP-9 increased. Of note, the increasing trend of the two serum markers was found significantly correlated. Trend of serum CRP was also very similar to that of IL-8 and MMP-9, but just below statistical significance. TGFβ1 levels were lower at stage III CRC, while IL-6 and VEGF levels had no significant variations. In tissue specimens, TGFβ1 receptors were already absent in about 50% of adenomas, and this percentage of missing receptors markedly increased in CRC stages III and IV.

**Conclusions:**

Combined quantification of serum IL-8, MMP-9 and CRP, appears a reliable and advanced index of inflammation-related processes during malignant phase of colorectal carcinogenesis, since these molecules remain within normal range in colorectal adenoma bearing patients, while consistently increase in the blood of CRC patients, even if from stage II only.

## Introduction

Colorectal carcinoma (CRC) is one of the most common malignant tumors worldwide, with one million new cases diagnosed each year. Almost 60% of cases occur in developed countries, and in Europe it is the second cause of cancer-related death [1]–[3]. CRC may develop from adenomatous polyps (adenomas), the precursor lesions, whose malignant potential depends on their size, morphology, and grade of dysplasia [4]. The fact that this type of malignant tumor commonly arises from adenomatous polyps has been proven in the case of hereditary colon cancer syndromes, which may carry up to 100% risk of carcinoma, as in familial adenomatous polyposis [5].

In the last few years, the role of inflammation in the initiation and progression of colorectal carcinoma has been investigated in greater depth. Inflammatory reactions are associated with almost all types of malignant tumors, and are known to promote tumor proliferation, angiogenesis and metastasis, and to increase resistance to hormonal or chemo-therapies. Monocyte/macrophage cells are usually the major component of the inflammatory infiltrate in the microenvironment of most malignant tumors; they are thus called tumor-associated macrophages (TAM). Phagocyte infiltration begins early in the non-invasive stage of the tumor, and continues progressively with an evident but gradual switch from the M1 pro-inflammatory phenotype to the M2 cancer-promoting phenotype. This event directly influences behavior and function of tumor cells, and promotes tissue remodeling and repair, immunomodulation, angiogenesis, and tumor progression [6]. Notably, colorectal cancer cells, stimulated by preexisting inflammation, or driven by intrinsic pathways related to gene defects, may also secrete different cytokines, and activate the local stromal cells to do likewise [7].

Among the cytokines involved in inflammation-associated intestinal tumorigenesis, an important role is played by interleukin-6 (IL-6) and interleukin-8 (IL-8). Both have been found over-expressed in CRC with increased angiogenic and metastatic potential [8]. The IL-6 and IL-8-dependent inflammatory network appears to significantly contribute to relating oncogene-induced cellular senescence with an inflammatory phenotype and tumor progression [9].

Other inflammation-related molecules, such as transforming growth factor β1 (TGFβ1), vascular endothelial growth factor (VEGF) and matrix metalloproteases (MMPs), become predominant during advanced tumor stages [6]. TGFβ1 is an important cytokine, which probably plays a major role in the association of inflammation and carcinogenesis. Under physiological conditions, this pleiotropic peptide is involved in regulating cell processes including proliferation, survival, differentiation and apoptosis: TGFβ1 inhibits the growth of epithelial cells, including intestinal epithelial cells, by enhancing differentiation and apoptotic pathways, while it promotes the proliferation of fibroblasts and myofibroblasts, as well as the deposition of extracellular matrix [10]. Further, TGFβ1 is an extremely potent immunosuppressive factor that inhibits proliferation, activation and differentiation of immune effector cells, thus its over-expression might contribute to promoting the invasive and metastatic behavior of tumor cells [11]. Indeed, in a previous study on a series of colorectal cancers, at different stages, from I to IV, the amount of TGFβ1 was found significantly increased only in the most advanced cancer stage [12].

Derangement of the TGFβ1 signal transduction pathway, mediated by mutations or polymorphisms of its receptors and/or of the transduction molecules, SMADs, is considered to play a primary role in the development and progression of several types of cancer in humans [13], [14]. In addition, the derangement of TGFβ1 receptors, TGFβ1RI or TGFβ1RII, can contribute to colon cancer formation and metastasis [15]. Our previous experience with a series of 15 patients, who had undergone CRC surgical resection, led to the observation of a significant reduction of TGFβ1RI (7/15) and TGFβ1RII (3/15) in the tumor mass versus the apparently normal surrounding colonic mucosa [12]. Further, mutations in the TGFβ1RII are estimated to occur in approximately 30% of CRC [15], [16].

On the basis of the current literature, sufficient data are now available concerning TGFβ1RI and TGFβ1RII changes in established CRC, but the trend of these molecules during the early phases of carcinogenesis, i.e. in benign tumors of the colorectum, remains to be defined.

VEGF has emerged as a critical mediator of angiogenesis, required for invasive tumor growth and metastasis and, therefore, targeted antibodies against VEGF and its receptors have been introduced as potential antitumor therapy [17]–[19]. Inflammatory cytokines themselves are potent activators of MMPs, enzymes that demolish extracellular matrix proteins and that are likely involved in all steps of colorectal carcinogenesis. However, notwithstanding their likely involvement in this process, the available data do not elucidate the actual trend of their serum levels during both benign and malignant phases of colorectal carcinogenesis [20]. The same applies to the levels of here investigated cytokines, which are considered as macrophage markers of phenotypes 1 and 2 by Mantovani and co-workers [21].

In this paper, a comprehensive group of patients affected by either benign (adenomas) or malignant (adenocarcinomas) colorectal tumors at different stages was investigated. The goal was: i) to define the trend of serum levels of IL-8, IL-6, TGFβ1, VEGF and MMPs, throughout the entire manifest process of colorectal carcinogenesis; ii) to compare the trend of the inflammatory cytokines IL-8 and IL-6 with that of C-reactive protein (CRP); iii) to characterize fully the behavior of TGFβ1RI and TGFβ1RII in tissue specimens from colorectal adenomas and different stages of adenocarcinomas.

## Patients and Methods

### Patient Recruitment

The study population comprised 96 patients affected by colonic adenoma or colorectal carcinoma, recruited at San Luigi Gonzaga University Hospital (Orbassano, Turin, Italy) between November 2008 and November 2010. Twenty-eight adenomas were endoscopically removed at the Division of Gastroenterology (9 women, 19 men, age range 47 to 79, median age 68 years). Sixty-eight CRC patients (31 women, 37 men, age range 46 to 80 years, median age 73) received surgery at the Division of General Surgery.

Of the colonic adenomas, based on the presence of villous structure, 11 were histologically classified as tubular adenomas (less than 20% of the tumor mass with villous structure) and 17 as tubulovillous adenomas (between 20 and 80% of the tumor mass with villous structure). Of the CRC patients, 11 were classified as stage I adenocarcinoma, 25 as stage II adenocarcinoma, 26 as stage III adenocarcinoma and 6 patients as stage IV adenocarcinoma, according to the TNM staging system. Patients were excluded from the study if they were aged above 80 or below 40 years, if they had associated chronic inflammatory diseases and/or previous surgical intervention on the intestine, or if they had received radiotherapy or chemotherapy before surgery. Seventeen healthy blood donors were selected as control group.

The study protocol, in agreement with the ethical guidelines of the 1975 Declaration of Helsinki, was approved by the local Ethical Committee (San Luigi Hospital, Orbassano, Italy, n. 191/INT) and written informed consent was obtained from each patient enrolled.

### Tissue Samples

Both adenoma biopsies and surgically-removed segments of CRC were fixed in 4% formaldehyde and embedded in paraffin for standard histological (hematoxylin-eosin staining) and immunohistochemical analyses.

### Blood Serum Samples

Blood specimens were taken from patients one day before surgical resection of the tumor mass, and on the day of the colorectoscopic procedure. The samples were collected in plain glass tubes and the serum was immediately separated by centrifugation (2000 g) then stored at −80°C, ready for analyses. In the control group, blood sampling, processing and storage were performed similarly.

### Detection of Inflammatory Molecules in Serum

Inflammatory molecules were detected using ELISA standard protocols and following the manufacturer’s instructions (ELISA ImmunoTools kit, Germany, for IL-6 evaluation; Instant ELISA kit, Bender Med System, Prodotti Gianni, Milan, Italy, for IL-8 evaluation; DuoSet ELISA kit, R&D Systems, UK, for TGFβ1, MMP-9 and VEGF_165,121,165b_ analyses). A 96-well microtiter plate reader (Model 680 Microplate Reader, Bio-Rad, Milan, Italy) was used to detect each molecule, and serum levels were calculated using the software SlideWrite (Advanced Graphics Software, CA, USA). IL-6, IL-8 and VEGF were expressed as pg/ml serum; TGFβ1 and MMP-9 were expressed as ng/ml serum.

### C-reactive Protein (CRP) Analysis

CRP was detected in the serum using a latex immunoassay. The CRP concentration was quantified by turbidimetry and the absorbance change was detected at 572 nm wavelength (Architect c System Analyzer, Abbott Diagnostics, Abbott Park, IL, USA). Serum levels of the protein were expressed in mg/L.

### Gelatin Zymography

MMP-2 and MMP-9 activities in serum samples were assayed by gel zymography. Proteins (100 µg) were separated by electrophoresis in 8% SDS-PAGE gel containing gelatin (0.8 mg/ml) under non-reducing conditions. The gel was washed with Tris buffer (2.5% Triton X-100 in 50 mM Tris-HCl, pH 7.5, final solution) for 1 hour and then incubated overnight at 37°C in a proteolysis buffer (40 mM Tris-HCl, 200 mM NaCl, 10 mM CaCl_2_, 0.02% NaN_3_, pH 7.5, final solution). The gel was then stained for 3 hours with Coomassie Blue solution (0.05% Coomassie Brilliant Blue R-250, 50% methanol, 10% acetic acid, final solution) and finally destained with 5% methanol and 7% acetic acid (final solution). Reagents and chemicals were from Sigma–Aldrich (Milan, Italy) and VWR International (Milan, Italy). MMP-9 activity was detected as a clear band on a blue background and estimated by densitometric analysis using Image J Software (USA). The results were expressed as percentages of control values.

### Immunohistochemical Analyses

TGFβ1RI, TGFβ1RII were tested on tumor tissue sections from all patients. Paraffin-embedded tissue was deparaffinized and rehydrated through graded alcohols. Heat-induced antigen retrieval was performed by microwave treatment in citrate buffer (pH 6.0) three times for 5 minutes, followed by cooling for 30 minutes at room temperature. Endogenous peroxidase activity was quenched by a 15 minute treatment with 3% hydrogen peroxide. Following 15 minute incubation in bovine serum in Tris-Buffered Saline (TBS)/Tween-20 solution, sections were incubated overnight in a humidified chamber at 4°C in the presence of specific polyclonal mouse antibodies (8 µg/ml anti-TGFβ1RI, 4 µg/ml anti-TGFβ1RII, Santa Cruz, Tebu-Bio s.r.l., Magenta, Milan, Italy). After washing with TBS, slides were incubated for 35 minutes with secondary anti-mouse antibodies (Dako, Glostrup, Denmark). The immune reaction was then revealed with EnVision System-HRP using diaminobenzidine as chromogen (Dako, Glostrup, Denmark). Sections were finally counterstained with hematoxylin, dehydrated through graded alcohols, mounted and examined at a Leica microscope (Leica Microsystems Wetzlar GmbH, Wetzlar, Germany). The staining intensity was evaluated by two independent observers, in random order, at 20x and 100x magnifications. Semiquantitative analysis was expressed as minimal, low, medium or high grade immunostaining as follows: the score was calculated as percentage of positive stained cells multiplied by corresponding staining intensity. This procedure determined a score of up to 12 points, which were categorized as minimal (score 1–3); low (score 4–6); medium (score 7–9) and high (score 10–12) reactivity.

In a limited number of sections from stage III colorectal cancers, the distribution of MMP-9 and IL-8 within the tumor mass was investigated. For this purpose, sections were incubated overnight in the presence of either 0.5 µg/ml anti IL-8 or 1 µg/ml anti MMP-9. The remaining technical procedure was the same as for immunohistochemical detection of TGFβ1 receptors (see above). The objectives used were 10x/0.22 and 40x/0.65.

### Statistical Analyses

Results were expressed as Mean ± Standard Error of the Mean (SEM). Statistical differences between groups were evaluated using Student’s t-test and one-way ANOVA test associated with the Bonferroni’s multiple comparison post test. To determine the degree of correlation among different serum variables of each patient within any single tumor group, non parametric Spearman’s test was performed. All data were analyzed with GraphPad InStat software (San Diego, USA).

## Results

### Serum Levels of IL-8, IL-6 and CRP in Patients Bearing Tubular or Tubulovillous Colorectal Adenoma or Adenocarcinoma at Different Stages of Progression

**Figure 1:**
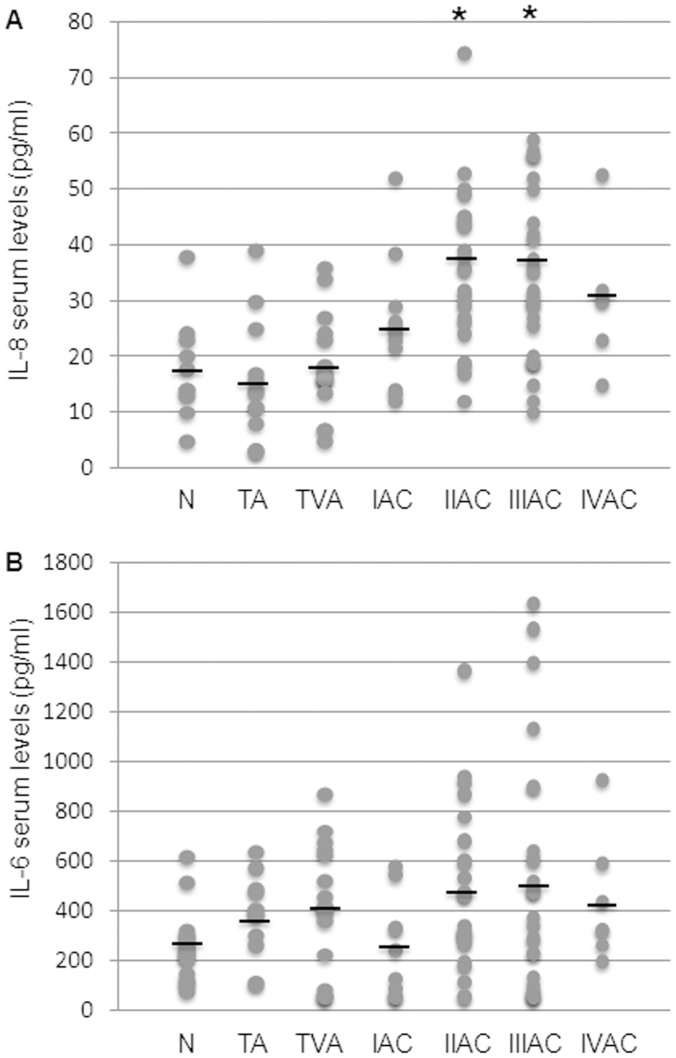
Evaluation of serum levels of IL-8 and IL-6 during colorectal carcinogenesis. The levels of IL-8 (A) and IL-6 (B) were measured in controls and patient groups (N: control; TA: tubular adenoma; TVA: tubulovillous adenoma; I-IV AC: malignant stages of adenocarcinoma). IL-8 and IL-6 were detected by ELISA and values expressed as pg/ml serum (see materials and methods). A: dots correspond to IL-8 single values and black lines represent the mean values within the experimental groups. Mean values ± SEM: N 17.0±2.5; TA 15.9±3.7; TVA 18.4±2.2; IAC 25.4±4.8; IIAC 38.7±5.1; IIIAC 37.2±5.7; IVAC 30.5±5.7. *Significantly different versus control group (p<0.05). B: dots correspond to single IL-6 values and black lines represent the mean values within experimental groups. Mean values ± SEM: N 247.1±55; TA 375.9±54; TVA 407.9±80; IAC 237.9±68; IIAC 472.3±89; IIIAC 521.3±152; IVAC 436.3±118. *Significantly different versus control group (p<0.05).

**Figure 2:**
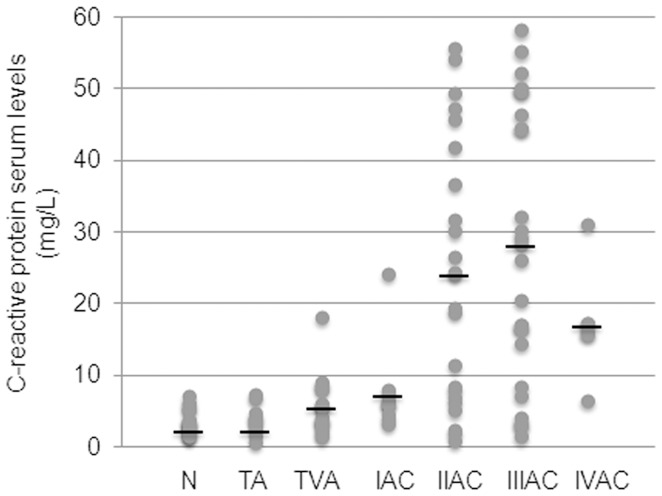
Evaluation of serum levels of CRP during colorectal carcinogenesis. The levels of CRP were measured in controls and patient groups (N: control; TA: tubular adenoma; TVA: tubulovillous adenoma; I-IV AC: malignant stages of adenocarcinoma). CRP was detected by latex immunoassay analysis and expressed as mg/L serum (see materials and methods). Dots correspond to single CRP values and black lines represent the mean values within the experimental groups. Mean values ± SEM: N 3.5±0.1; TA 3.7±0.7; TVA 5.7±1.1; IAC 7.5±1.8; IIAC 24.3±8.5; IIIAC 27.9±7.5; IVAC 17.2±4.1.

Figure 1 shows levels of IL-8 and IL-6, as markers of the pro-inflammatory macrophage phenotype, in the peripheral blood of patients undergoing endoscopic or surgical resection, of colorectal adenoma or adenocarcinoma, respectively. IL-8 serum levels remained within the control range in patients affected by colonic tubular or tubulovillous adenoma. The IL-8 level then began to rise in stage I CRC patients, showing a statistically significant increase in those with cancer at stages II and III, i.e. the two largest groups under investigation (Figure 1A). Unlike IL-8, serum levels of IL-6 in tumor-bearing patients did not show any significant changes versus controls, although a modest but inconsistent increase was observed at cancer stages II and III (Figure 1B). Notably, in the same cohort of patients, CRP, i.e. the most commonly used inflammatory marker, showed a trend quite similar to that of IL-8, with normal serum levels in patients with benign colorectal tumors, and an increase starting from stage II of malignancy, but just below statistical significance (Figure 2).

### Serum Levels of TGFβ1 and VEGF in Patients Bearing Tubular or Tubulovillous Colorectal Adenoma or Adenocarcinoma at Different Stages of Progression

**Figure 3:**
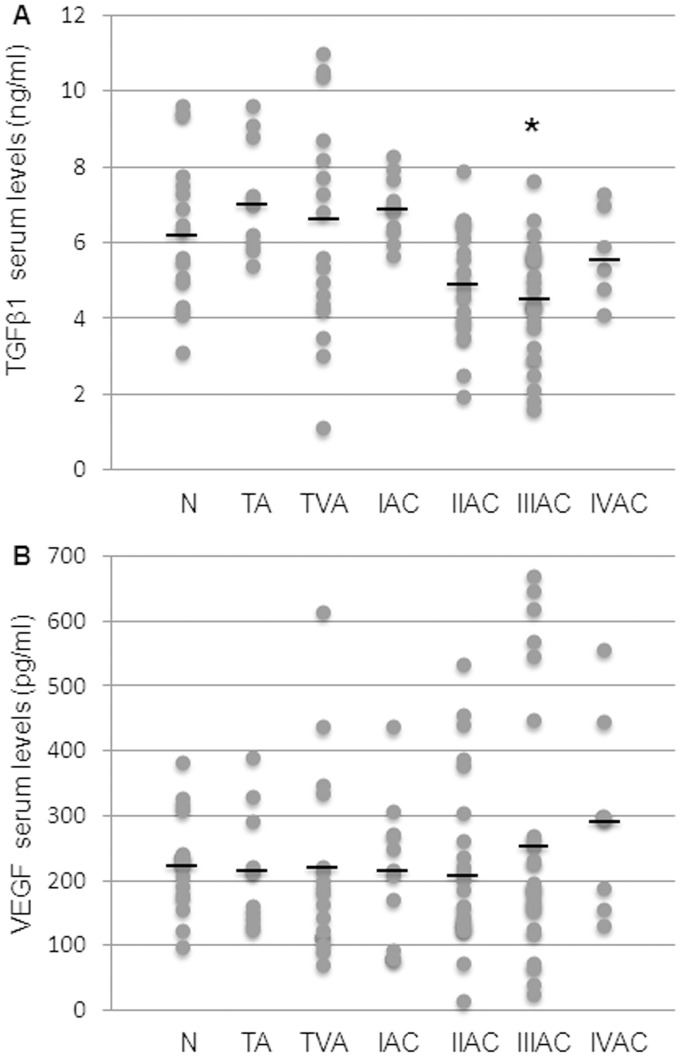
Evaluation of serum levels of TGFβ1 and VEGF during colorectal carcinogenesis. The levels of TGFβ1 (A) and VEGF (B) were measured in controls and patient groups (N: control; TA: tubular adenoma; TVA: tubulovillous adenoma; I-IV AC: malignant stages of adenocarcinoma). TGFβ1 and VEGF were detected by ELISA and values expressed as ng/ml and pg/ml serum, respectively (see materials and methods). A: dots correspond to single TGFβ1 values and black lines represent the mean values within the experimental groups. Mean values ± SEM: N 6.3±0.4; TA 7.2±0.45; TVA 6.3±0.6; IAC 6.9±0.3; IIAC 4.9±0.3; IIIAC 4.4±0.3; IVAC 5.7±0.5. *Significantly different versus control group (p<0.05). B: dots correspond to single VEGF values and black lines represent the mean values within the experimental groups. Mean values ± SEM: N 227.0±18.7; TA 215.2±31.3; TVA 221.1±36.4; IAC 216.3±34.4; IIAC 205.0±26.6; IIIAC 266.5±38.6; IVAC 295.2±70.2.

In the same cohort of oncologic patients and healthy controls, the serum concentrations of TGFβ1 and VEGF, were determined. The mean serum levels of the pleiotropic cytokine TGFβ1, measured in patients with either tubular or tubulovillous colorectal adenomas, as well as in patients with stage I adenocarcinoma, did not show any significant variation versus controls. The TGFβ1 serum levels decreased at stages II and III adenocarcinomas, although only at stage III adenocarcinoma a significant difference was achieved versus controls (Figure 3A).

Conversely, there was no evident variation in VEGF levels in the peripheral blood of controls versus patients affected by either adenomas or adenocarcinomas, although a slight but not significant increase was observed at advanced stages of adenocarcinoma (Figure 3B).

### Immunohistochemical Analysis of TGFβ1RI and TGFβ1RII in Specimens of Tubular or Tubulovillous Colorectal Adenoma or Adenocarcinoma at Different Stages of Progression

As there is considerable evidence suggesting that tumor cells may become not responsive to TGFβ1-dependent cell signaling, the levels and distribution of the two main receptors of TGFβ1 were analyzed in histological tissue sections of adenomas and adenocarcinomas. Semi-quantitative analyses were performed, applying a commonly-used immunostaining score. Peritumoral colonic mucosa had a uniform staining for both TGFβ1RI and TGFβ1RII. Unexpectedly, at the early steps of colorectal carcinogenesis, i.e. in tubular and tubulovillous adenomas, the amount of RI and RII receptors expressed by the neoplastic cells was below that of normal mucosa in about 50% of the specimens examined, with one or both receptors reduced in each tumor specimen. Quite similar results were obtained for TGFβ1RI and/or TGFβ1RII levels in stage I and II adenocarcinomas, with decreased receptor levels in 60% and 56% of the cases, respectively. At the more advanced stages of colorectal cancer progression, expression of TGFβ1 receptors in tumor tissue showed a clear-cut decrease in the vast majority of stage III and in all stage IV adenocarcinomas (Table 1).

**Table 1:** Analysis of the two main TGFβ1 receptors in tissue samples of adenomas and adenocarcinomas.

	Patients with decreased levels of TGFβ1 receptors	Type of receptors altered (number of cases)		
		TGFβ1RI	TGFβ1RII	TGFβ1RI & TGFβ1RII
TA	12/21 (57%)	–	4	8
TVA	10/20 (50%)	–	–	10
IAC	3/5 (60%)	1	2	–
IIAC	5/9 (56%)	1	2	2
IIIAC	7/10 (70%)	2	2	3
IVAC	7/7 (100%)	2	3	2

TGFβ1RI: TGFβ1 receptor I; TGFβ1RII: TGFβ1 receptor II.

TA: tubular adenoma; TVA: tubulovillous adenoma; I-IV AC: malignant stages of adenocarcinoma.

In brackets percentages of patients with alteration of the TGFβ1 receptor system. Minimal or low grade immunostaining was taken as an index of receptors’alteration.

### Metalloprotease Activity and MMP-9 Protein Levels in Serum of Patients Bearing Tubular or Tubulovillous Colorectal Adenoma or Adenocarcinoma at Different Stages of Progression

**Figure 4:**
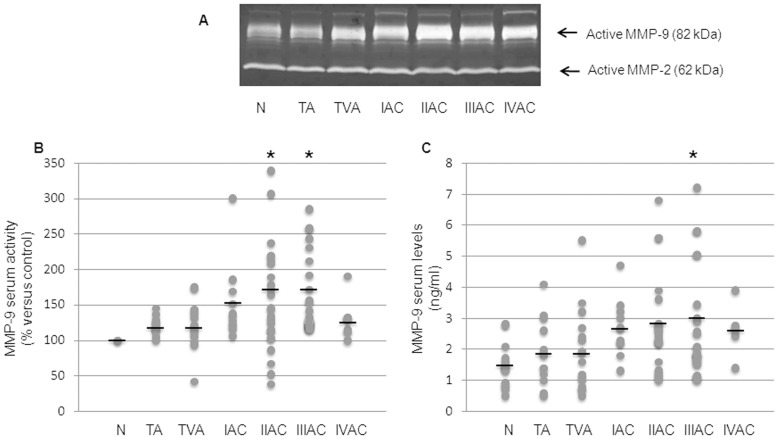
Evaluation of serum MMP activity and levels during colorectal carcinogenesis. A: active forms of MMP-2 and MMP-9 are shown in a representative gelatin zymography. B: MMP-9 activation in the serum was evaluated by densitometric analysis as percentage of active MMP-9 compared to controls (taken as 100%). Dots correspond to the activated MMP-9 value for each subject and black lines represent mean values within the experimental groups. Mean values ± SEM: TA 120±3.9; TVA 121±8.5; IAC 151.63±17.2; IIAC 163.81±16.6; IIIAC 164.2±10.6; IVAC 129.0±13.0. *Significantly different versus control group (p<0.01). C: serum levels of MMP-9 protein were detected by ELISA and expressed as ng/ml serum. Dots correspond to the MMP-9 value for each subject and black lines represent the mean values within the experimental groups. Mean values ± SEM: N 1.5±0.2; TA 1.9±0.3; TVA 1.9±0.3; I AC 2.7±0.3; IIAC 2.8±0.3; IIIAC 3.0±0.5; IVAC 2.6±0.4. *Significantly different versus control group (p<0.05). N: control; TA: tubular adenoma; TVA: tubulovillous adenoma; I-IV AC: malignant stages of adenocarcinoma.

The activity of both the metalloproteases principally involved in malignant phenotype acquisition, i.e. MMP-2 and MMP-9, was measured by zymography. While MMP-2 activity showed no significant difference at any tumor stage (Figure 4A), the behavior of MMP-9 was more interesting: the enzymatic activity increased in malignant tumors, being significantly different at stages II and III of cancer progression (Figure 4A/B).

Based on these observations, we decided to quantify serum levels of MMP-9 as a further considered marker of type 2 monocytic activation. In line with observations for MMP-9 activity, serum protein levels showed a net and significant increase in the malignant phases of colorectal carcinogenesis, with mean values significantly different from controls at stage III (Figure 4C). Notably, at this stage of progression the increase of MMP-9 significantly correlated with IL-8 serum levels (Spearman’s rank correlation coefficient r = 0.4331, p = 0.039).

### Immunohistochemical Analysis of IL-8 and MMP-9 in Specimens of Adenocarcinoma at Advanced Stage of Progression

**Figure 5:**
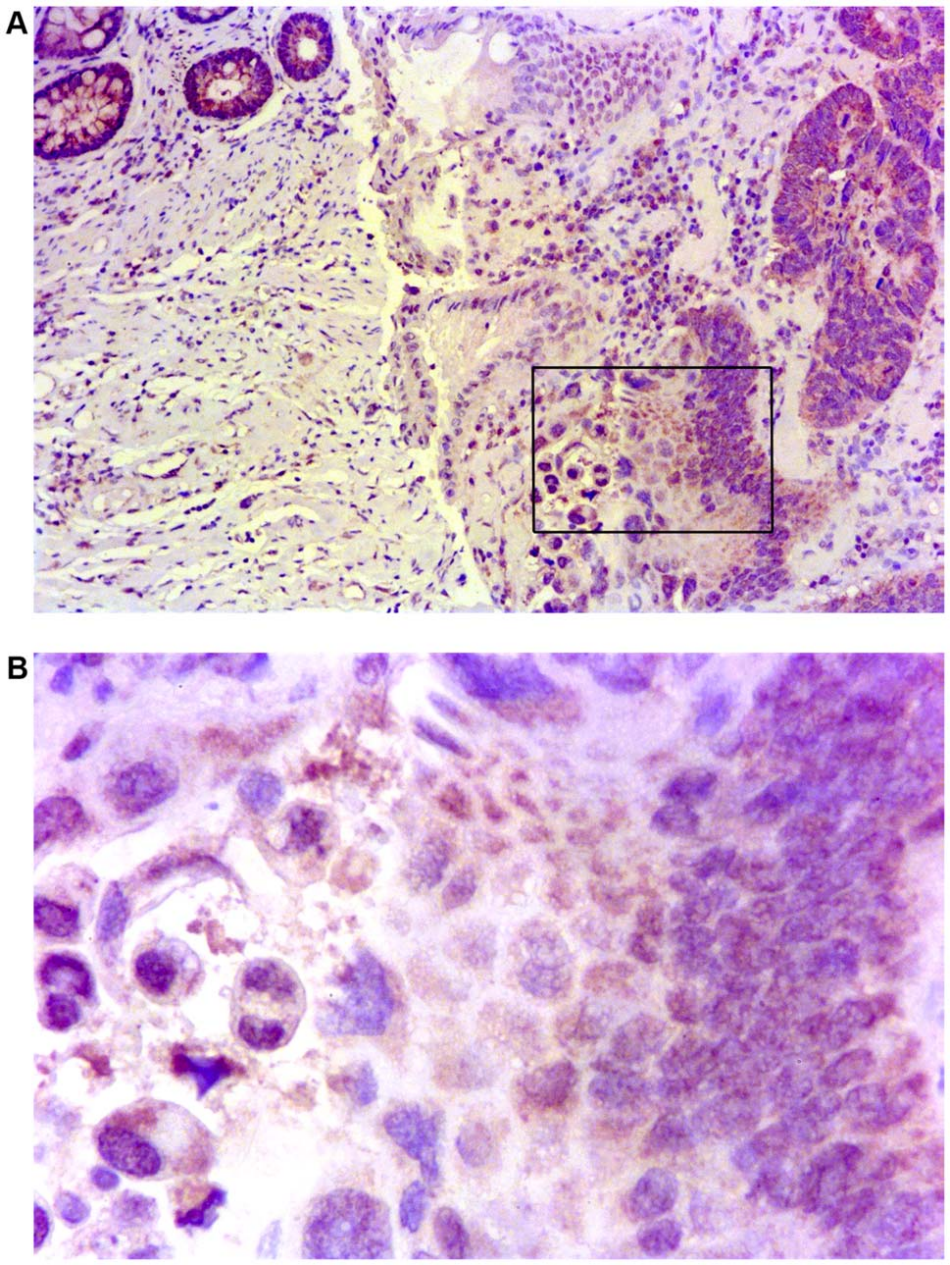
Distribution and expression of MMP-9 on paraffin embedded CRC tissue sections. A: a representative image of carcinoma tissue at stage III showed strong staining for MMP-9, localized in the cytoplasm of both tumor cells and inflammatory cells (10x/0.22 objective). For experimental details see materials and methods. B: higher magnification of a MMP-9 positive area of the same cancer section (40x/0.65 objective). The images were obtained using Digital Microscope DMD Leica, Leica Microsystems, Milan, Italy.

Taking into account the statistically significant increase of IL-8 and MMP-9 concentrations in the peripheral blood of patients at stage II and III adenocarcinoma, the actual distribution of these two molecules was then verified in a limited number of corresponding CRC sections by immunohistochemistry. While IL-8 resulted to be expressed mainly by inflammatory cells infiltrating the adenocarcinoma in advanced stage of progression, and apparently not by cancer cells (data not shown), MMP-9 was clearly produced in large amount by both macrophages and tumor cells (Figure 5).

## Discussion

This study provides a first overall picture of the trend of serum inflammation markers, during the entire manifest phases of colorectal carcinogenesis, namely in benign adenomas and in the different stages of CRC. To date, relevant studies have focused on the malignant phases of this multistep process; many have been less than comprehensive, and have not investigated trends of potential serum markers of inflammation and disease progression during the expression of benign lesions, i.e. colorectal adenomas.

The proinflammatory cytokines IL-6 and IL-8 are known to be involved in the development of colorectal cancer [22]–[24]. Tumor-associated macrophages are the most likely source of these cytokines, but colon cancer cells themselves were reported to synthesize IL-8, under inflammatory conditions [8], [25]–[27]. In our study, the immunohistochemical analysis of CRC specimens did not show significant presence of IL-8 within cancer cells or the surrounding stroma, being the cytokine production mainly dependent on the inflammatory infiltrate (data not shown). Further, the observed trend of IL-8 levels in the peripheral blood of patients bearing colorectal adenomas and adenocarcinomas clearly demonstrates that the increase of this important inflammatory mediator is limited to the malignant phase of the neoplastic disease (Figure 1A). More precisely, serum IL-8 began to significantly increase at stage II adenocarcinoma. Hence, although inflammatory reactions were certainly triggered by the developing neoplasia well before reaching stage II, net evidence of inflammation occurred in the serum relatively late during the multistep process of colon carcinogenesis, and IL-8 alone can not be taken as a specific marker of malignancy in the neoplastic disease of colorectum. Contrary to the behavior of IL-8, IL-6 did not show any particular variation in the serum of cancer patients versus healthy individuals (Figure 1B).

In parallel with these cytokines, serum levels of CRP, which is routinely used as an inflammation marker, were analyzed. CRP displayed a trend very similar to that of IL-8 serum levels (Figure 2). Whereas this provides further support for the validity of CRP as a general marker of inflammation, it also strongly suggests that CRP analysis should be combined with that of other inflammatory molecules, as it has already been recommended [28]. An appropriate spectrum of inflammatory cytokines, including IL-8, might be considered as a potential tool for monitoring inflammation in the serum of patients with CRC.

Very recently, a multiplex-based analysis of the major inflammatory cytokines and growth factors considered to be potential serum markers for colon cancer pointed to IL-8 as the only molecule of this class that might be useful for diagnosis and disease monitoring. The inflammation-related molecules simultaneously quantified in 100 serum samples of colon cancer patients (stages I to IV) were: IL-1α, IL-1β, IL-2, IL-4, IL-6, IL-8, IL-10, VEGF, EGF, TNFα, IFNγ, MCP-1, but only IL-8 serum levels showed a statistically significant difference between controls and adenocarcinoma patients. Further, in full agreement with our findings, the serum IL-8 increase was evident at stages II, III, IV of colorectal cancer, while in the blood of patients with stage I adenocarcinoma it was still within the control range [29]. None of the other cytokines and growth factors tested discriminated between controls and cancer patients. In this context, mean serum VEGF values increased in cancer patients versus controls, but the study failed to find any statistical significance [29].

The participation of angiogenesis in the later phases of colorectal tumor progression has been widely described, and connections have been reported between the levels of VEGF expression and tumor invasivity and aggressiveness [30]–[32]. However, in our series of patients, mean serum VEGF levels showed no significant change during the progression from benign to malignant colorectal neoplastic disease, probably because our data were more widely dispersed. In addition, the small number of patients at the advanced stage of CRC is a limiting factor in our study (Figure 3B).

As regards TGFβ1 serum levels, a negative trend was observed at stages II and III adenocarcinomas, with a significant difference only at stage III (Figure 3A). According to the theory now widely recognized, loss of susceptibility to the differentiating and pro-apoptotic effects of TGFβ1 provides a major escape mechanism favoring malignancy. For instance, not only the low concentration of circulating TGFβ1, but also the alteration of its signaling pathway, may concur in tumor development and progression [12], [15], [33]. For this reason, although the stated aim of our study was to monitor serum inflammation markers during the entire process of colorectal carcinogenesis, we exploited the availability of colonic adenoma biopsies to obtain information on the expression of TGFβ1RI and/or TGFβ1RII in the benign phase of the carcinogenic process. Our results are quantitatively limited, but may well be the first available data of such type. The semiquantitative immunohistochemical measurement of TGFβ1 receptors in our series of benign colorectal tumor specimens indicates that derangement of TGFβ1RI and/or TGFβ1RII may already be evident at the benign step of colonic carcinogenesis (Table 1). If this finding is confirmed, it would mean that the loss of susceptibility to the anti-proliferative effect of this cytokine is not *per se* sufficient to explain the shift from benign to malignant phenotype, and thus other genetic and/or epigenetic alterations would be necessary for the progression to cancer to occur.

MMP-9 is an enzyme recognized to be primarily involved in the formation of metastases. Serum levels of MMP-9 were found to be elevated in many studies on CRC and our results confirmed those reports [34], [35]. The increase of mean serum levels of MMP-9 (Figure 4B) was statistically significant in stage III adenocarcinoma patients and was also found to be significantly correlated with increased serum levels of IL-8 of CRC at stage III. Thus, serum MMP-9 could be another valid marker of colorectal cancer progression, especially if combined with IL-8, (and CRP). Further, immunohistochemical staining of MMP-9 in CRC specimens showed strong positivity in tumor cells and confirmed that tumor associated macrophages are important source of MMPs during the carcinogenic process (Figure 5).

Analysis of MMP-9 activation in the serum by zymography provided findings of particular interest. Serum levels of activated MMP-9 showed a trend that was strikingly similar to that of the IL-8 (Figure 4A). As found with IL-8, mean serum values of activated MMP-9 in patients with tubular or tubulovillous adenomas, as well as those at stage I adenocarcinoma, consistently remained within the control range. Both parameters only increased significantly from stage II adenocarcinoma onwards. Thus, if these findings should be confirmed by additional data, they would indicate that the two different macrophage phenotypes, so named M1 and M2, may be present simultaneously in overt colorectal cancer, rather than there being a distinct switch from the M1 pro-inflammatory phenotype to the M2 tumor-promoting phenotype, as in the hypothesis put forth by Mantovani and Sica [6].

In conclusion, we investigated the trend of blood levels of IL-8, IL-6, TGFβ1, VEGF and MMP-9, throughout the benign to malignant phases of colorectal neoplastic disease. IL-8 levels and MMP-9 activity and levels showed very similar trends, increasing concomitantly and only in patients with established cancer, starting from stage II adenocarcinoma. An almost identical trend was exhibited by the common inflammatory marker CRP. These findings point to the combined quantification of IL-8, MMP-9 and CRP as a reliable and advanced index of inflammation-related processes occurring in the malignant phase of colorectal carcinogenesis. Most likely, all such parameters drop dramatically after surgical removal of the malignant neoplasia and may rise again in case of disease’s relapse. Follow-up of the patients recruited in this study is in progress to confirm this hypothesis and to verify if a direct correlation exists between IL-8/MMP-9 increased levels and poor clinical outcome.
